# Induction of Heterosubtypic Cross-Protection against Influenza by a Whole Inactivated Virus Vaccine: The Role of Viral Membrane Fusion Activity

**DOI:** 10.1371/journal.pone.0030898

**Published:** 2012-01-27

**Authors:** Natalija Budimir, Anke Huckriede, Tjarko Meijerhof, Louis Boon, Emma Gostick, David A. Price, Jan Wilschut, Aalzen de Haan

**Affiliations:** 1 Department of Medical Microbiology, Molecular Virology Section, University Medical Center of Groningen, Groningen, The Netherlands; 2 Bioceros, Utrecht, The Netherlands; 3 Department of Infection, Immunity & Biochemistry, Cardiff University School of Medicine, Cardiff, United Kingdom; Hallym University, Republic of Korea

## Abstract

**Background:**

The inability of seasonal influenza vaccines to effectively protect against infection with antigenically drifted viruses or newly emerging pandemic viruses underlines the need for development of cross-reactive influenza vaccines that induce immunity against a variety of virus subtypes. Therefore, potential cross-protective vaccines, e.g., whole inactivated virus (WIV) vaccine, that can target conserved internal antigens such as the nucleoprotein (NP) and/or matrix protein (M1) need to be explored.

**Methodology/Principal Findings:**

In the current study we show that a WIV vaccine, through induction of cross-protective cytotoxic T lymphocytes (CTLs), protects mice from heterosubtypic infection. This protection was abrogated after depletion of CD8+ cells in vaccinated mice, indicating that CTLs were the primary mediators of protection. Previously, we have shown that different procedures used for virus inactivation influence optimal activation of CTLs by WIV, most likely by affecting the membrane fusion properties of the virus. Specifically, inactivation with formalin (FA) severely compromises fusion activity of the virus, while inactivation with β-propiolactone (BPL) preserves fusion activity. Here, we demonstrate that vaccination of mice with BPL-inactivated H5N1 WIV vaccine induces solid protection from lethal heterosubtypic H1N1 challenge. By contrast, vaccination with FA-inactivated WIV, while preventing death after lethal challenge, failed to protect against development of disease and severe body weight loss. Vaccination with BPL-inactivated WIV, compared to FA-inactivated WIV, induced higher levels of specific CD8+ T cells in blood, spleen and lungs, and a higher production of granzyme B in the lungs upon H1N1 virus challenge.

**Conclusion/Significance:**

The results underline the potential use of WIV as a cross-protective influenza vaccine candidate. However, careful choice of the virus inactivation procedure is important to retain membrane fusion activity and full immunogenicity of the vaccine.

## Introduction

Influenza represents one of the major health burdens worldwide [1]. Although vaccination is the cornerstone of protection against influenza, currently used seasonal vaccines elicit a narrow strain-specific antibody response that neutralizes antigenically matched virus strains, but fails to protect against antigenically drifted strains or newly emerging pandemics viruses [2], [3]. Protection against different influenza virus subtypes and variants requires the development of vaccines that are capable of inducing heterosubtypic immunity [4]. Such vaccines should target not only the variable surface antigen of the virus, hemagglutinin (HA), but also more conserved internal antigens, such as the nucleoprotein (NP) and/or matrix protein (M1) [5], [6].

One strategy to induce heterosubtypic immunity is vaccination with a formulation that has the capacity to induce cross-reactive CD8+ cytotoxic T lymphocyte (CTL) responses against conserved antigens shared by different influenza virus subtypes [5], [7], [8]. CTL-mediated heterosubtypic immunity, although unable to neutralize the virus and prevent infection, could facilitate clearance of the virus, thereby controlling the course of infection [5], [9].

Previously, we reported that vaccination of mice with whole inactivated virus (WIV) induces activation of naive and primed CTLs specific for NP [10]. Importantly, the activation of such CTLs, especially the priming of naive cells, was influenced by the way in which the virus was inactivated. This was most likely due to differential effects of the inactivation procedure on viral membrane fusion properties. It has been suggested that inactivation of some viruses, such as Rift Valley Fever virus or Respiratory Syncytial Virus, using formalin (FA) or β-propiolactone (BPL) can induce distortion of antibody epitopes or suppress antigen processing [11], [12]. Interestingly, we showed that inactivation of influenza virus using FA severely compromises the membrane fusion activity of WIV. In contrast, inactivation using BPL largely preserves viral membrane fusion activity. Moreover, vaccination with FA-inactivated WIV (FA-WIV) primes naive CD8+ T cells less effectively than vaccination with BPL-inactivated WIV (BPL-WIV). After receptor-mediated endocytosis of WIV particles by antigen-presenting cells (APC), membrane fusion activity most likely facilitates the “escape” of viral antigens from endosomes into the cytosol. Here influenza antigens can be processed and cross-presented to CD8+ T cells in an MHC class I-restricted manner [13], [14], [15].

In the current study, we investigate whether immunization of mice with WIV (H5N1) protects against lethal heterologous challenge (H1N1). We specifically investigated the extent to which such protection might depend on the inactivation procedure used in the production of WIV and, consequently, membrane fusion activity. The cross-protective capacity of WIV was compared directly with standard inactivated influenza vaccines, both subunit and split.

Mice vaccinated with BPL-WIV were protected against lethal heterologous challenge and the development of disease symptoms. This cross-protection was mediated by CD8+ cytotoxic T cells. By contrast, vaccination with FA-WIV, although protecting from virus-induced death, failed to prevent severe body weight loss associated with decrease in daily activity. Mice vaccinated with subunit or split vaccine did not survive lethal challenge with heterologous virus. These data demonstrate that vaccination with WIV, but not subunit or split vaccine, induces cross-protection against lethal heterologous infection. Importantly, our findings highlight the impact of virus inactivation protocols on the overall immunogenicity of the vaccine formulation and indicate that the preservation of membrane fusion activity is critical for optimal efficacy.

## Results

### Heterosubtypic cross-protection induced by vaccination with WIV

To investigate the capacity of influenza WIV vaccine to induce cross-protective immunity, we vaccinated mice with WIV derived from NIBRG-14 virus, a reassortant containing the surface antigens of A/Vietnam/1194/2004 (H5N1) virus and the internal core of A/PR/8/34 (H1N1) virus, and subsequently challenged the animals with PR/8 virus. The extent to which cross-protection depends on the mode of virus inactivation was assessed by comparing FA-WIV with BPL-WIV. FA-WIV was produced by incubation of NIBRG-14 influenza virus with 0.01% FA for 7 days at 4°C [16]. BPL-WIV was produced by incubation of NIBRG-14 virus with 0.1% BPL for 24 hr at 4°C. After either inactivation protocol, viral replicative capacity was completely destroyed (Fig. S1).

C57Bl/6 mice were immunized twice with FA-WIV or BPL-WIV subcutaneously (s.c.) at a dose equivalent to 6 µg of viral HA. Two other groups of mice were vaccinated with subunit or split vaccine, and a control group was mock-vaccinated with vehicle buffer. After the second immunization, animals were exposed to a lethal total respiratory tract challenge with 100 PFU (corresponding to 2×10^2^ TCID_50_) of live PR/8 virus. All mice were monitored for body weight change, and euthanized when their body weight loss exceeded 20%.

**Figure 1:**
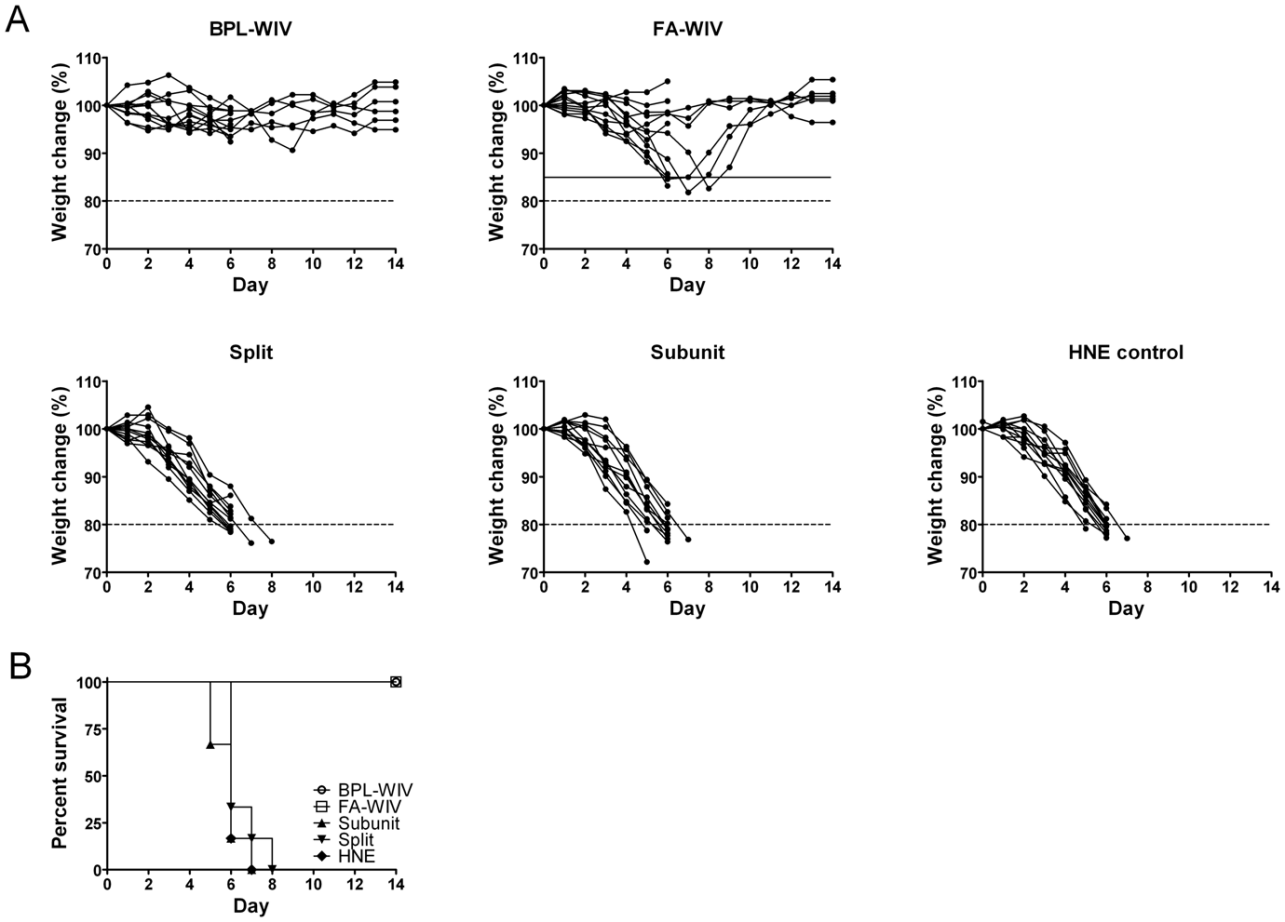
Body weight change and survival of vaccinated mice after heterologous challenge. Mice were vaccinated twice with vaccines derived from H5N1 virus (BPL-WIV, FA-WIV, split, subunit) or mock-vaccinated with HNE, and then challenged with H1N1 virus. (A) After challenge, animals were monitored daily for body weight change. Body weight loss of more than 15% (solid line) associated with decline in daily activity was considered to represent severe symptoms. Body weight loss of more than 20% was an indication for euthanasia (dashed line). Each group contained 12 animals, 6 of which were followed up for 6 days and then sacrificed for the measurement of immune response parameters; the other 6 mice were monitored for 14 days. A higher proportion of animals vaccinated with FA-WIV exhibited significant body weight loss and severe disease symptoms compared to animals vaccinated with BPL-WIV (p<0.05; Fisher's exact test). (B) All mice vaccinated with either FA-WIV or BPL-WIV survived the heterologous challenge based on a body weight loss of less than 20%, as indicated above. In contrast, mock-vaccinated mice or mice that received either the split or subunit vaccines did not survive the challenge.

Over the course of 14 days post-challenge, only mice vaccinated with BPL-WIV survived lethal heterosubtypic challenge without body weight loss or apparent symptoms of disease (Fig. 1a and 1b). By contrast, in the group of mice vaccinated with FA-WIV, 5 out of 12 animals developed severe disease symptoms combined with more than 15% body weight loss. By the end of the follow-up period, these mice slowly recovered and regained their normal body weight. This difference in the quality of protection induced by vaccination with BPL-WIV or FA-WIV was statistically significant by Fisher's exact test (Fig. 1a). Mice vaccinated with subunit or split vaccine were not protected from heterosubtypic challenge. Similar to mock-vaccinated mice, these animals lost more than 20% of their body weight within 7 days post-challenge, and all of them were euthanized on the basis of pre-established criteria by day 8 (Fig. 1a and 1b).

**Figure 2:**
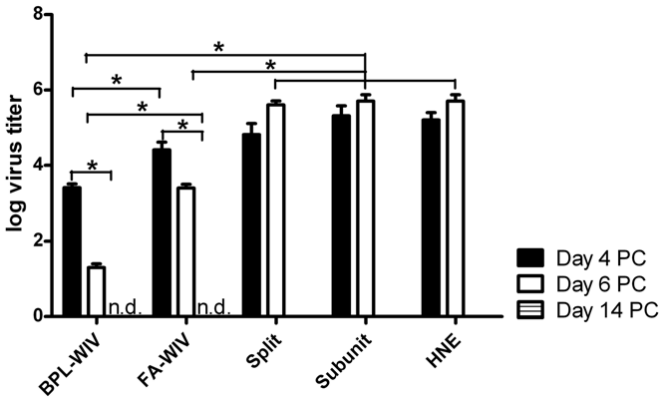
Virus titers in the lungs of vaccinated mice after heterologous challenge. Virus titers in the lungs of mice vaccinated with the indicated formulations were measured at days 4 and 6 after challenge. Bars represent mean titer±SEM of three mice. Mice vaccinated with either BPL-WIV or FA-WIV had lower virus titers in the lungs at both days 4 and 6 compared to the titers in mice vaccinated with split vaccine, subunit vaccine or HNE buffer (p<0.05 for all comparisons; Mann-Whitney U test). Furthermore, in mice vaccinated with WIV, virus titers were lower at day 6 compared to titers at day 4 post-challenge (p<0.05; Mann-Whitney U test). Finally, significantly lower titers were observed in mice vaccinated with BPL-WIV compared to titers in mice vaccinated with FA-WIV, both at day 4 and day 6 (p<0.05; Mann-Whitney U test). No virus was detected in surviving mice at day 14 after challenge (n.d., not detectable).

Next, we assessed the capacity of BPL-WIV or FA-WIV to induce clearance of influenza virus from the lungs of challenged mice. Although both vaccines led to improved virus clearance, the dynamics of clearance were significantly different for the two vaccination groups. On day 4 post-challenge, mice immunized with BPL-WIV had lower virus titers in the lungs compared to mice immunized with FA-WIV. By day 6, mice in both vaccination groups had lower virus titers compared to those measured on day 4. Again, the titers in mice vaccinated with BPL-WIV were significantly lower compared to the titers in mice vaccinated with FA-WIV. Virus titers in the lungs of mock-vaccinated mice and mice vaccinated with either split or subunit vaccines remained high on both days (Fig. 2).

### Role of antibodies in cross-protection induced by vaccination with WIV

**Figure 3:**
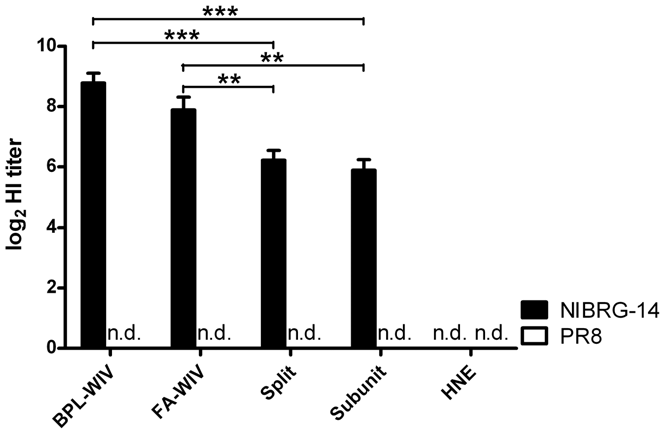
H5N1- and H1N1-specific HI antibodies measured in pre-challenge sera of vaccinated mice. Bars indicate the geometric mean titers±SEM of 9 mice per vaccination group. HI antibodies specific for H5N1 were detected in all vaccination groups (BPL-WIV, FA-WIV, subunit and split), with significantly higher titers measured in mice vaccinated with BPL- and FA-WIV. In contrast, HI antibodies against PR/8 (H1N1) were not observed in any of the mice (n.d., not detectable).

The results presented above show that vaccination of mice with BPL-WIV derived from H5N1 virus induced better protection from disease symptoms and more rapid clearance of heterosubtypic H1N1 virus than vaccination with FA-WIV, split or subunit vaccine derived from the same H5N1 virus. To rule out the possibility that this difference was due to the more efficient induction of antibodies against HA by BPL-WIV, we determined the hemagglutination-inhibition (HI) titers against the vaccine (H5N1) and challenge (H1N1) viruses induced by these vaccine formulations. Pre-challenge sera of immunized mice were collected and tested for the level of HI antibodies. BPL-WIV and FA-WIV induced similar levels of HI antibodies against the vaccine (H5N1) strain, which were significantly higher compared to titers induced by split and subunit vaccine. In contrast, neither of the vaccines induced detectable levels of HI antibodies cross-reactive with the challenge (H1N1) strain (Fig. 3). This finding implies that antibodies detectable by HI assay most likely did not contribute significantly to the observed cross-protection (Fig. 3).

**Figure 4:**
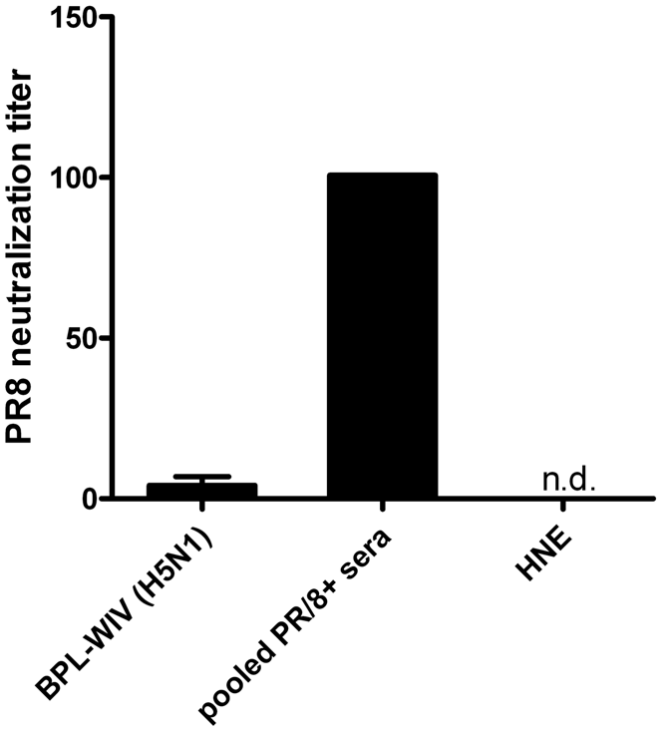
H1N1-neutralization antibodies measured in pre-challenge sera of mice vaccinated with H5N1 BPL-WIV. Bars indicate the geometric mean titers±SEM of 6 mice in the H5N1 BPL-WIV vaccination group or the titer measured in the pooled sera from 10 mice vaccinated with H1N1 PR/8-derived vaccine and challenged with PR/8 virus. Sera from mice vaccinated with H5N1 BPL-WIV showed very low neutralization activity against H1N1 PR/8.

Cross-protection observed after vaccination with BPL-WIV could be due to the activity of neutralizing antibodies that are incapable of inhibiting hemagglutination but are capable of binding to, for example, the stem region and/or fusion peptide of HA, thus preventing HA-mediated membrane fusion and infection. We therefore performed PR/8 microneutralization assay to screen for the existence of possible cross-neutralizing antibodies in pre-challenge sera of mice vaccinated with H5N1 BPL-WIV. Figure 4 shows that sera from mice immunized with H5N1 BPL-WIV have only a minimal capacity to neutralize PR/8 virus when compared to sera from mice immunized with PR/8-derived vaccine and challenged with PR/8 virus. This suggests that cross-reactive antibodies do not play a major role in the observed protection induced by vaccination with BPL-WIV.

### Role of CD8+ T cells in cross-protection induced by vaccination with WIV

**Figure 5:**
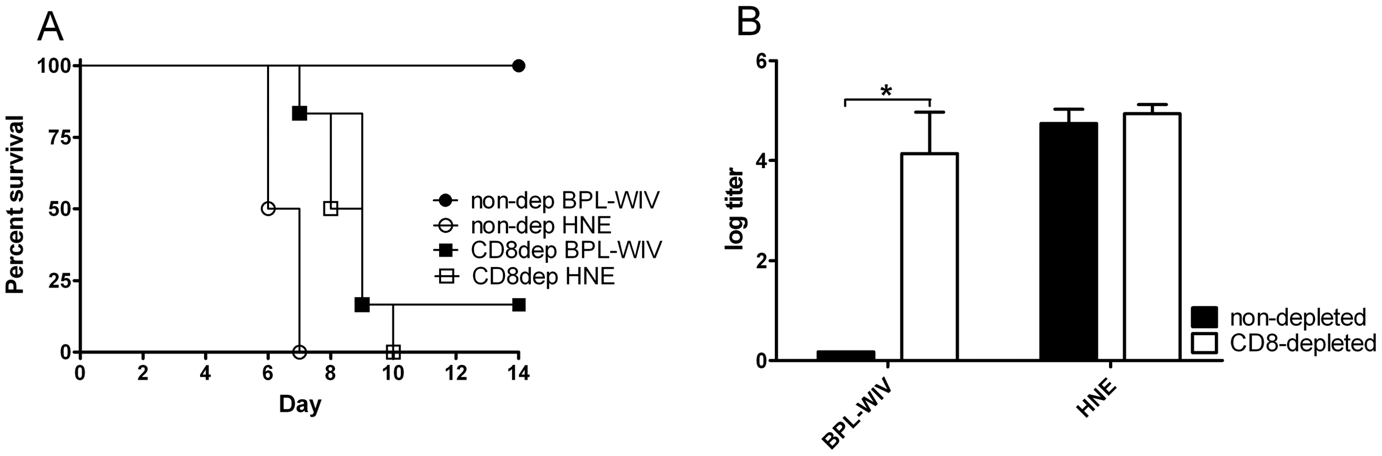
Survival and reduction in lung virus titers after heterologous challenge depends on the presence of CD8+ cells. Mice were vaccinated with H5N1-derived BPL-WIV or mock-vaccinated with HNE. Before heterologous lethal challenge, half of the animals (n = 6) from each vaccination group were randomly selected and received CD8+ cell depletion antibody treatment, as described in the Materials and Methods. (A) All mice vaccinated with BPL-WIV survived the heterologous challenge, based on a body weight loss of less than 20%. In contrast, protection was lost in CD8+ cell-depleted mice and survival rates were comparable to those of non-immune mice. (B) At the time of sacrifice, high levels of virus were detected in the lungs of vaccinated CD8+ cell-depleted mice, similar to those observed in non-immune mice. At the same time point post-challenge, virus could not be detected in the lungs of mice vaccinated with BPL-WIV in the absence of CD8+ cell depletion (n.d., not detectable).

To investigate whether the observed cross-protection induced by immunization with BPL-WIV was mediated by CD8+ CTLs, we depleted CD8+ cells from mice immunized with BPL-WIV by intraperitoneal (i.p) administration of CD8+ cell depletion antibody on three consecutive days immediately prior to challenge, with two additional doses administered post-challenge. In peripheral blood (Fig. S2) and spleen (data not shown), >98% of CD8+ cells were depleted. Comparable to mock-vaccinated mice, BPL-WIV-vaccinated mice were not protected against lethal heterosubtypic challenge after depletion of CD8+ T cells (Fig. 5a). By day 9 post-challenge, most of these mice exhibited more than 20% body weight loss and had to be euthanized. Virus titers measured in the lungs of these animals were high and did not differ from the titers observed in the lungs of mock-vaccinated mice (Fig. 5b). These data indicate that the cross-protection induced by vaccination with WIV is mediated primarily by CD8+ CTLs.

### Induction of CTL responses by BPL-WIV and FA-WIV: the role of membrane fusion activity

**Figure 6:**
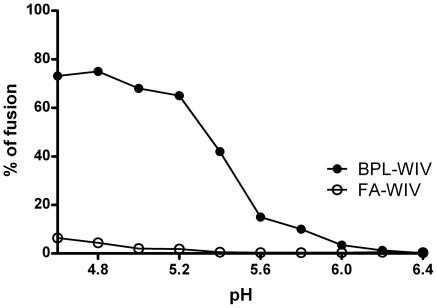
Effect of virus inactivation treatments on the membrane fusion activity of WIV formulations. Guinea pig erythrocytes were incubated with BPL-WIV or FA-WIV at different pH values for measurement of pH-dependent viral membrane fusion. The amount of released hemoglobin was measured by spectrophotometry at 540 nm. Release of hemoglobin expressed relative to maximal release of hemoglobin in water was used as a measure of fusion. Only BPL-WIV showed the capacity to fuse to erythrocyte membranes when incubated at low pH (4.6–5.6), as shown by the amount of released hemoglobin. In contrast, in the same pH range, no fusion of FA-WIV with erythrocyte membranes was detected.

Previously, we showed that FA-treatment abolishes the membrane fusion activity of WIV and that such a formulation has a decreased capacity to prime CTL activity in mice [10]. Here, we first confirmed that the FA-WIV used in the present study completely lacked membrane fusion activity as assessed on the basis of hemolysis activity (Fig. 6). By contrast, up to 80% of the membrane fusion activity was preserved in the BPL-WIV preparation (Fig. 6). Importantly, the difference in hemolysis induced by BPL-WIV or FA-WIV was not due to a difference in erythrocyte binding, as determined by hemagglutination assay (data not shown).

**Figure 7:**
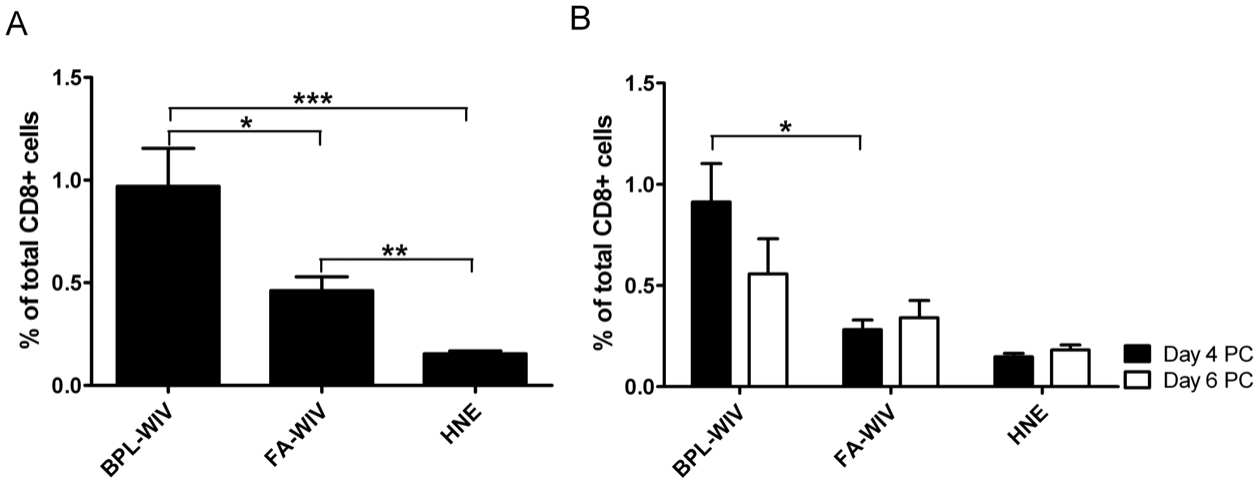
BPL-WIV induces higher levels of NP-specific CD8+ T cells compared to FA-WIV. (A) Numbers of influenza NP_366–374_-specific CD8+ T cells were determined before challenge in the peripheral blood of mock-vaccinated mice and mice vaccinated with either BPL-WIV or FA-WIV. Bars indicate the mean percentage±SEM of 9 mice per group. Vaccination of mice with BPL-WIV induced significantly higher levels of NP_366–374_-specific CD8+ T cells compared to levels induced by FA-WIV (p<0.05; Mann-Whitney U test). (B) Four days after challenge, the mean percentage of influenza NP_366–374_-specific CD8+ T-cells in the spleen was significantly higher in mice vaccinated with BPL-WIV compared to mice vaccinated with FA-WIV (p<0.05; Mann-Whitney U test, n = 3).

Next, we compared the levels of influenza-specific CTLs induced by FA-WIV and BPL-WIV in peripheral blood and spleen. In PBMCs from immunized mice, pre-challenge, CTLs specific for influenza NP_366–374_ were detected with cognate MHC class I tetramers in both groups of mice (Fig. 7a). However, the level of NP-specific CTLs was significantly higher in mice vaccinated with fusion-active BPL-WIV compared to mice vaccinated with fusion-inactive FA-WIV. Notably, an early accumulation of NP-specific CTLs in the spleen was observed 4 days post-challenge in mice vaccinated with fusion-active BPL-WIV, but not in mice receiving fusion-inactive FA-WIV (Fig. 7b). By day 6 post-challenge, this population decreased slightly.

### Influx of specific CTLs into the lungs of WIV-vaccinated mice after infection

**Figure 8:**
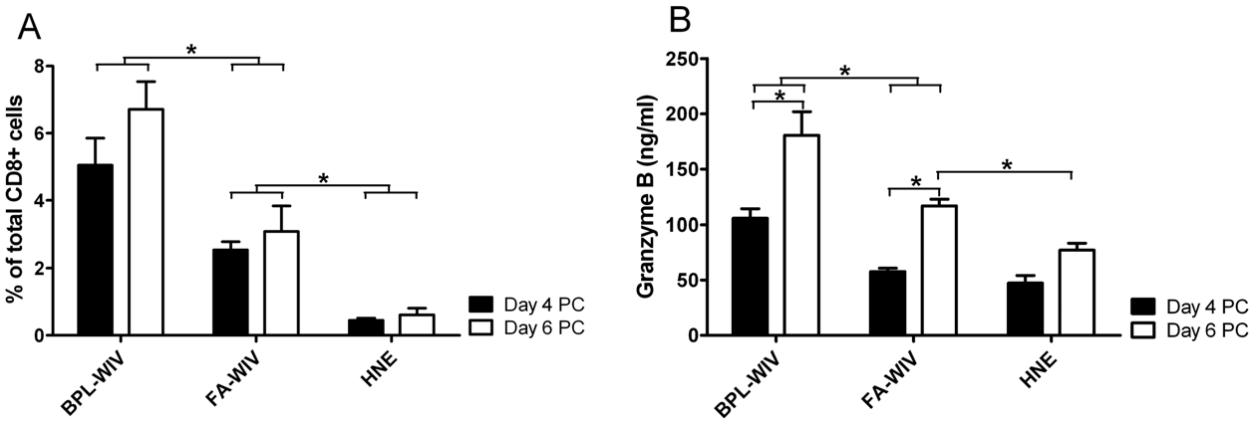
BPL-WIV vaccination results in a higher post-challenge influx of cytotoxic CD8+ T cells into the lungs. (A) The accumulation of NP_366–374_-specific CD8+ T cells in the lungs of mice vaccinated with BPL-WIV or FA-WIV was measured on days 4 and 6 post-challenge. Bars indicate the mean percentage±SEM of 3 mice per group. On days 4 and 6 post-challenge, significantly higher percentages of NP_366–374_-specific CD8+ T cells were observed in mice vaccinated with BPL-WIV compared to mice vaccinated with FA-WIV (p<0.05; Mann-Whitney U test). (B) The higher influx of NP_366–374_-specific CD8+ T cells in BPL-WIV vaccinated mice was accompanied by a higher local production of granzyme B compared to levels measured in mice vaccinated with FA-WIV (p<0.05; Mann-Whitney U test). In both the BPL-WIV and FA-WIV vaccinated groups, levels of granzyme-B were significantly enhanced on day 6 compared to levels measured on day 4 (p<0.05; Mann-Whitney U test).

Finally, we investigated whether the superior protection induced by fusion-active BPL-WIV, compared to fusion-inactive FA-WIV, was associated with a higher influx of cytotoxic CD8+ T cells into the lungs as the site of infection. Lung-associated lymphocytes were isolated from mice euthanized on day 4 or 6 post-challenge and influenza NP_366–374_-specific CTLs were enumerated by tetramer staining. Additionally, bronchoalveolar lavages (BALs) were collected to measure the level of locally produced granzyme B as an indicator of cytotoxic effector activity. On day 4 and 6 post-challenge, higher levels of NP-specific CTLs were detected in the lungs of mice vaccinated with fusion-active BPL-WIV compared to mice vaccinated with fusion-inactive FA-WIV (Fig. 8a). At the same time, local granzyme B levels were significantly higher in mice vaccinated with fusion-active BPL-WIV compared to the levels observed in mice vaccinated with fusion-inactive FA-WIV (Fig. 8b). Thus, the influx of NP-specific CTLs in the lungs and local production of granzyme B correlated with early clearance of influenza virus from the lungs of mice vaccinated with fusion-active BPL-WIV.

## Discussion

In this study, we show that vaccination of mice with a whole inactivated influenza virus vaccine induces cross-protection against heterosubtypic challenge, in contrast to vaccination with either subunit or split vaccines. Importantly, we demonstrate that the procedure used for virus inactivation in the production of WIV influences the magnitude of the immune response, and consequently the extent of cross-protection. Subunit and split vaccines proved unable to induce influenza-specific CTL activity. In contrast, vaccination with BPL-WIV induced a strong CTL response associated with rapid clearance of virus from the lungs of challenged mice and an apparent absence of disease symptoms. In comparison, vaccination with FA-WIV induced lower levels of CTLs associated with a slower rate of virus clearance and the development of disease symptoms that gradually regressed by the end of the follow-up period. These differences in the magnitude of the CTL response and the efficacy of cross-protection observed after vaccination with the different WIV preparations were linked to the effects of the inactivation procedures on viral membrane fusion activity.

The observed cross-protection induced by vaccination with WIV appears to be mediated primarily by influenza-specific CTL activity. Indeed, depletion of CD8+ cells in vaccinated mice abrogated cross-protection against heterosubtypic challenge. This rules out the possibility that anti-HA antibodies play a major role. Accordingly, cross-reactive HI antibodies specific for the challenge (H1N1) virus were undetectable in the H5N1-vaccinated mice. Recent reports have shown that antibodies against the conserved stem region of HA or against neuraminidase (NA) display cross-reactivity among different influenza subtypes [17], [18], [19], and these antibodies would not be detected in standard HI assays. Microneutralization assays could, however, identify these antibodies. In our hands sera of mice immunized with H5N1 WIV showed minimal A/PR/8/34 virus-neutralizing capacity *in* vitro. Although low levels of cross-reactive antibodies and, possibly cross-protective CD4+ T cells [20], may thus be elicited by vaccination with WIV, these effector mechanisms apparently are not strong enough in isolation to substantially suppress viral replication, as shown in vaccinated mice with depleted CD8+ cells. Thus, specific CTLs appear to mediate the observed cross-protection of WIV-vaccinated mice against heterosubtypic challenge.

The difference in cross-protection induced by WIV on one hand, and by subunit and split vaccines on the other, is correlated to the (in)ability of these vaccines to prime specific CTL activity. A major requirement for the induction of influenza-specific CTLs is the presence of conserved viral antigens in the vaccine, as CTL responses are mainly directed against internal conserved proteins [21], [22], [23]. Additional characteristics that may contribute to the induction of CTL activity by WIV include the particulate structure of the vaccine, which enables efficient uptake of vaccine antigens by dendritic cells (DCs) through the process of receptor-mediated endocytosis [24]. Finally, WIV contains viral genomic ssRNA, which acts as an intrinsic adjuvant, activating TLR7-signaling in DCs [25]. This ssRNA-induced TLR7-signaling has been shown to contribute to superior activation of the humoral immune response by WIV [26]. Likewise, we have experimental evidence indicating that priming of CTL activity by WIV critically depends on TLR7-signaling (Budimir *et al.*, manuscript in preparation). The inability to induce cross-protective CTL activity is not surprising for the subunit vaccine. Indeed, being composed of isolated viral surface antigens, the subunit vaccine does not contain internal CTL antigens. On the other hand, the split vaccine does contain all the protein components of the influenza virus, yet in our studies it failed to induce cross-protective CTL activity. Being composed of non-particulate antigen, split vaccine may not be taken up and processed efficiently by DCs [27]. Wagner *et al.* showed that the induction of cross-priming required insertion of split vaccine antigens into microspheres, facilitating targeting of the antigens to the endosomes of DCs [26]. Also, the split vaccine lacks intact immunostimulatory viral ssRNA [26], which, as indicated above, contributes to the ability of WIV to prime CTLs. It has been shown that coupling of split influenza vaccine antigen to an alternative immunostimulatory TLR-ligand, CpG, boosts the priming of CTL activity [28]. Thus, it appears that the specific intrinsic properties of the vaccine formulation, such as composition and structure, determine its capacity to induce cross-protective CTL responses.

WIV as a non-replicating vaccine presumably activates target CTLs through cross-priming, mediated by professional APCs, particularly DCs [29]–[32]. After being taken up by DCs, vaccine antigens end up in the endosomal compartment. From there, these antigens have to “escape” to the cytosol where they can enter the MHC class I antigen presentation pathway. Indeed, Bender *et al.* have demonstrated that the delivery of WIV-derived antigen to the cytosol of APCs is essential for successful priming of CTLs [33]. Intact membrane fusion activity of WIV most likely facilitates “escape” of internal viral antigen from the endosome to the APC cytosol, where it can be processed and presented to CD8+ cytotoxic T cells through the MHC class I pathway. Previously, we observed that inactivation of membrane fusion activity of WIV diminishes its capacity to prime specific CTL activity [10]. Here, we show that fusion-active WIV induces superior heterosubtypic cross-protection in mice compared to fusion-inactive WIV. This indicates that activation of CTLs through cross-presentation of WIV-derived antigens depends on the fusogenic properties of the vaccine.

Some procedures used for virus inactivation can severely impair the membrane fusion properties of the virus, thereby affecting the capacity of the vaccine to induce CTL activity. In this study, we have shown that an inactivation protocol, relying on treatment of influenza virus with formaldehyde (FA), completely abolishes membrane fusion activity of the virus. This protocol is commonly used by vaccine manufacturers to generate WIV [16]. In contrast, virus inactivation based on treatment with β-propiolactone (BPL) largely preserves membrane fusion activity. In our studies, only vaccination with BPL-WIV prevented severe body weight loss and the development of disease symptoms by inducing optimal CTL activity in the lungs and rapid clearance of the virus (Ref [10]; Fig 1a, 2, 7a). By contrast, vaccination with FA-WIV gave only partial protection, with 5 of 12 vaccinated mice showing severe body weight loss and disease symptoms. This suboptimal cross-protection was associated with a comparatively low CTL response. Our findings therefore imply that preservation of virus membrane fusion activity should be taken into account when WIV vaccines are generated.

The important role of CTLs specific for conserved influenza antigens, such as NP, in protection against influenza infection in humans and mice has been recognized and reported earlier [5], [34]. In the present study, we show that vaccination with WIV induces high levels of specific CTLs in blood and spleen. After heterologous challenge, these CTLs infiltrate the lungs, where they are already detectable by day 4 post-infection. These findings are in line with those of Fonteneau *et al.*, who showed that WIV can induce *in vitro* proliferation of influenza-specific CD8+ T cells in humans [29]. Critically, we also show that CTLs induced by vaccination with WIV protect mice from lethal heterosubtypic challenge. In a recent study, Furuya *et al.* showed that immunization of mice with WIV produced using a different inactivation method (γ-irradiation) also protects mice against lethal infection with heterologous virus [35]. By contrast, Bodewes *et al.* reported that immunization with WIV induced poor activation of CTLs and a low level of cross-protection [36]. The discrepancy between our results and those of Bodewes *et al.* could be explained on two levels. First, Bodewes *et al.* used an FA-based protocol for inactivation of the virus, which interferes with optimal priming of CTL activity, as discussed above. Second, the difference in the route of administration used by us and Bodewes *et al.* could be an explanation for the different observations. In our experiments, the vaccine was administered through the s.c. route, while Bodewes *et al.* used i.m. injection. Several studies have demonstrated that the route of antigen administration can affect the magnitude of CTL activation as well as the diversity of the induced CTL response [37], [38]. For example, Combadiere *et al.* showed that transcutaneous application of influenza vaccine to humans induced better CD8+ T cell responses, in terms of both magnitude and quality, than injection though the i.m. route [37].

The inability of seasonal influenza vaccines to protect against drifted or newly emerging, potentially pandemic, viruses underlines the need for broadly cross-protective influenza vaccines. Clearly, induction of immunity against conserved viral antigens is crucial for-cross protection [6], [39], [40]. In this respect, there are specific lessons to be learned from the recent swine-origin H1N1 pandemic. Unexpectedly, the virus induced relatively mild disease [41]. Although cross-reactive antibodies may have contributed to the protection against the pandemic virus [18], [39], recent studies have suggested that the development of severe disease and mortality were largely prevented by existing memory CD8+ cytotoxic T cells specific for highly conserved virus proteins, such as NP and M1, induced by a prior infection with epidemic H1N1 virus [42]. In addition, other studies showed that human memory CD8+ T cells cross-react to a high extent with conserved NP and M1 epitopes from the highly pathogenic avian influenza H5N1 virus [43], [44]. In the present study, we demonstrated the cross-protective potential of properly inactivated influenza WIV, using a combination of NIBRG-14 (H5N1) vaccine and A/PR/8/34 (H1N1) challenge virus, which share internal antigens, including NP. Although this may favor an optimal response to challenge with the heterosubtypic virus, published data point to the high degree of internal antigen conservation, not only among different human influenza virus strains or subtypes but also between human and zoonotic viruses. In addition, it has been demonstrated that CTLs against internal viral antigens display a certain level of cross-reactivity to non-identical antigens [42]–[44]. Although not capable of preventing the viral infection itself, such CTL-mediated immunity can control the course of the disease by keeping the viral load suppressed [5], [6].

Although a renewed interest for WIV as a candidate cross-protective influenza vaccine has emerged, concerns about its reactogenicity remain. It is of interest to note, however, that this reactogenicity is not a uniform finding for all previously tested WIV vaccines and, additionally, also appears to relate to other factors such as vaccine mass and HA content [45]. With respect to the latter, the use of HA content for dosing of WIV vaccines gave a better correlate for reactogenicity than did chicken-cell agglutination, an older test for standardizing antigenic content of influenza vaccines [45]. Thus, improved analysis and standardization of WIV vaccines could reduce its reactogenicity.

In summary, we show here that one of the oldest influenza vaccines, WIV, induces heterosubtypic cross-protection that is closely dependent on specific CTL activity. Importantly, the capacity of WIV to induce an optimal CTL response, and consequently cross-protection, is strongly influenced by the procedure used for virus inactivation. WIV is therefore a promising candidate cross-protective influenza vaccine provided that suitable inactivation procedures, which retain viral fusion activity, are employed.

## Materials and Methods

### Ethics statement

All mouse experiments were performed in strict accordance with Dutch legislation on animal experiments (“Wet op de dierproeven”, 1977; modified in 1996 with implementation of the European guidelines 86/609/EEG and “Dierproevenbesluit 1985”) and approved by the Ethics Committee on Animal Research of the University Medical Center Groningen (Permit number: 5101).

### Virus strains and vaccines

H5N1 virus (NIBRG-14, a 6∶2 reassortant strain of A/PR/8/34 and A/Vietnam/1194/2004) cultured on Madine-Darby Kidney (MDCK) cells and egg-derived H1N1 virus (PR/8/34) were a kind gift from Solvay Biologicals (Weesp, The Netherlands). NIBRG-14 virus was inactivated and processed to different vaccine formulations: WIV, split and subunit. WIV vaccine was prepared either by 24 hr inactivation of the virus with 0.1% BPL (Acros Organics, Geel, Belgium) at room temperature, followed by dialysis for 24 hr against HNE buffer (5 mM HEPES, 150 mM NaCl, 0.1 mM EDTA, pH 7.4), or by incubation for 7 days in presence of 0.01% FA, with constant, mild stirring, followed by dialysis for 24 hr against HNE.

Inactivation of the vaccines was tested by performing serial passages on eggs, according to the protocol published in the European Pharmacopeia [46]. Specifically, one vaccine dose (20 µg of total viral protein) was inoculated into the allantoic cavity of each of 20 fertilized eggs and incubated at 33°C for 3 days. As a replication-positive control, 1 hemagglutination unit (HAU) of live NIBRG-14 virus was injected into the eggs. Portions of 1 ml of allantoic fluid from all the eggs were pooled, and 200 µl was inoculated into new eggs. This passage procedure was repeated one more time. Finally, after the last passage, allantoic fluid was harvested and tested for the presence of replicative virus by a hemagglutination test, as described elsewhere [47].

Fusion activity of WIV preparations was assessed using a hemolysis assay, as described previously [10].

BPL-WIV was processed to split vaccine according to a published protocol [48]. Subunit vaccine was prepared by processing BPL-WIV as described previously [49].

The protein content of all vaccine preparations was determined using the Bio-Rad protein assay kit (Bio-Rad Laboratories, Veenendaal, The Netherlands).

### Vaccination and challenge

Female C57Bl/6 mice, aged 6–8 weeks, were purchased from Harlan, The Netherlands. Every vaccination group contained 18 mice and was randomly divided into 3 subgroups that were analyzed for viral titers and immune response parameters at days 4, 6 and 14 post-challenge, respectively. Mice were vaccinated twice subcutaneously (s.c.) on days 0 and 21. Animals from all vaccination groups received an equal amount of 2×6.7 µg HA per vaccine dose, corresponding to 2×20 µg of total protein in the case of the split and WIV vaccines [50]. One week after the booster immunization, mice were anesthetized and inoculated intranasally with 100 PFU (corresponding to 2×10^2^ TCID_50_) of PR/8 (H1N1) in 40 µl HNE. A dose of 100 PFU was used because this was the minimal dose which reproducibly induced lethal infection in 100% mice. Following challenge, mice were monitored daily for body weight change. Body weight loss of more than 15% associated with decline in daily activity was considered to represent severe symptoms. Body weight loss of more than 20% was considered an indication for euthanasia. On day 4 and 6 post-challenge, 6 mice from each vaccination group were euthanized and blood, spleen, lungs and bronchoalveolar lavage (BAL) were collected. Mice that did not undergo more than 20% body weight loss were euthanized on day 14 post-challenge for analysis of virus titers and different immunological parameters.

Vaccination, blood sampling, challenge and euthanasia were performed under isoflurane anesthesia.

### Depletion of CD8+ cells

Depletion of CD8+ cells was performed by administration of purified CD8-specific depleting monoclonal antibody (clone YTS169). On days 22, 23 and 24 after the start of the experiment, mice were injected i.p. with 200 µg of the depletion antibody. Further, starting from day 1 post-challenge, mice received a single i.p. injection of the depletion antibody every 7 days. The efficacy of CD8+ cell depletion throughout the experiment was determined by staining of PBMCs with anti-mouse CD8a antibody (Immunosource, Zoersel, Belgium) and flow cytometry analysis. Blood samples were taken, and PBMCs isolated each time before injection of the depletion antibody dose.

### Lung virus titer measurements

On days 4, 6 and 14 post-challenge, the lungs of mice were dissected and collected in 1 ml phosphate buffered saline (PBS) on ice, homogenized mechanically and centrifuged for 10 min at 350×g. Supernatants were collected, snap-frozen and stored at −80°C until further processing.

For viral titrations, MDCK cells were seeded in flat-bottom 96-wells plates and incubated in serum-free Episerf medium (Invitrogen, Leek, The Netherlands) for 2 days in a CO_2_ incubator (37°C, 5% CO_2_). Two-fold dilutions of lung supernatants were then prepared in duplicate and 100 µl of each dilution was added to the MDCK cells, followed by a 1 h incubation at 37°C, 5% CO_2_. After incubation, the culture medium was replaced by medium supplemented with 6 µg/ml TPCK trypsine (Sigma-Aldrich, Zwijndrecht, The Netherlands) per well, and cells were incubated for additional 72 hr. On day 7, cell supernatants were harvested and transferred to V-bottom 96-wells plates. The presence of virus was detected using the hemagglutination assay [47].

### Tetramer staining of blood, spleen and lung lymphocytes

Pre-challenge blood samples were collected on the day of challenge by performing orbital puncture under isoflurane anesthesia. Samples were collected in tubes previously coated with heparin and incubated for 5 min with ACK buffer (0.15 M NH_4_Cl, 10 mM KHCO_3_, 0.1 mM EDTA) to induce lysis of erythrocytes. After 3 washes with FACS buffer (1% BSA, 5 mM EDTA in PBS), cells were stained with anti-mouse CD8a-APC antibody and influenza NP_366–374_-tetramer-PE. Soluble, biotinylated AM9/H-2Db monomeric protein was produced and tetramerized at a 4∶1 molar ratio with PE-conjugated streptavidin as described previously [51]. Dead cells were excluded using 7AAD viability solution (Immunosource). Flow cytometric analysis was performed using a FACSCalibur flow cytometer (BD Biosciences).

Spleens were dissected and collected on ice in Iscove's Modified Dulbeco's Modified Eagle's (IMDM) medium (Invitrogen) supplemented with 10% fetal bovine serum (FBS), 1% antibiotics and 0.1% β-mercaptoethanol. Splenocytes were isolated by homogenizing spleens though cell strainers (BD Bioscinece, Breda, The Netherlands) and resuspended in medium. After 10 min centrifugation (350×g) at 4°C, erythrocytes were removed by lysis using ACK buffer (0.15 M NH_4_Cl, 10 mM KHC0_3_, 0.1 mM Na_2_EDTA, pH 7.2). Finally, after an additional round of washing in medium, cells were washed 3 times in FACS buffer and stained according to the procedure described above.

Tetramer staining on lung derived lymphocytes was performed as follows. Mice were anesthetized and lungs were perfused through the heart with a total of 20 ml of PBS with heparin. After perfusion, lungs were dissected and cut into small pieces using a sterile scalpel, while cooled on ice. The pieces of lung tissue were incubated at 37°C in presence of 1 mg/ml collagenase D (Roche, Woerden, The Netherlands) for 3 hours. Next, lung homogenates were forced through cell strainers (BD Biosciences) and washed 3 times with Dulbeco's Modified Eagle's medium (DMEM; PAA, Colbe, Germany) supplemented with 2% FBS. Finally, lymphocytes were isolated using lympholyte density gradients (Sanbio, Uden, The Netherlands) according to the manufacturer's protocol. After washing in FACS buffer, cells were stained as described above.

### Granzyme B ELISA

Granzyme B production in the lungs of mice was measured in BAL samples by ELISA (R&D Systems, Abingdon, United Kingdom). Before testing, samples were centrifuged at 350×g for 10 min and supernatants were harvested. Five different dilutions were then prepared: a non-diluted sample, and 1∶10, 1∶50, 1∶100 and 1∶1000 diluted samples. The assay was performed according to the manufacturer's protocol.

### Hemagglutination inhibition (HI) assay

Serial two-fold dilutions of NIBRG-14 and PR/8 virus stocks were prepared in duplicates in V-bottom 96-wells plates. A suspension of 1% guinea pig erythrocytes was added and gently mixed with diluted virus. After incubation for 2 hr at room temperature, 1 HAU was scored as the highest virus dilution at which total hemagglutination was obtained. For determination of the HI titer, pre-challenge sera of vaccinated mice were complement-inactivated by incubating 75 µl aliquots for 30 min at 56°C. Next, sera were mixed with 3 volumes (225 µl) of 25% kaolin solution, incubated for 20 min at room temperature and centrifuged for 2 min at 400×g. Supernatants were collected and serially diluted two-fold in duplicate in V-bottom 96-wells plates. The virus suspension containing 4 HAU (determined as described above) and 1% guinea pig erythrocytes were then gently mixed with the serum supernatants. Hemagglutination was allowed to develop for 2 hr, and the highest dilution at which hemagglutination was inhibited was scored and used to calculate titers.

### Microneutralization assay

MDCK cells were seeded in 96-wells flat-bottom plates and cultured overnight in serum-free Episerf medium. The next day, two-fold serial dilutions of sera, with starting dilution 1∶10, were prepared in 96-wells plates in quadruplicate. To each well, with the exception of negative controls, 50 TCID_50_ of the challenge PR/8 virus was added and plates were incubated for 2 hours at 37°C. Next, MDCK cells were washed once with Episerf medium, and mixtures of serum and virus were added to the cells. Further on, cells were incubated for 1 hr at 37°C. After the 1 hr incubation, the culture supernatants were replaced by medium supplemented with 6 µg/ml of TPCK trypsine and cells were incubated for an additional 72 hr. On day 5, cell supernatants were harvested and transferred to V-bottom 96-wells plates. The presence of virus was detected using a hemagglutination assay [47].

### Statistical methods

Differences between vaccination groups with regard to the levels of tetramer-positive cells, granzyme B production, HI and lung virus titers were analyzed using the Mann-Whitney U test with a confidence interval 95%. To enumerate the difference between groups based on severe body weight loss, a Fisher's exact test was used. A value of p<0.05 was considered as statistically significant and is indicated in the figures with an asterisk. Double and triple asterisks indicate p values of <0.01 and <0.001, respectively.

## Supporting Information

Figure S1 **Assessment of viral inactivation status in the FA-WIV and BPL-WIV vaccines.** Influenza virus inactivation was tested by performing serial passages on eggs. After the last passage, allantoic fluids were tested for the presence of replicative virus using the hemagglutination test. Virus titers measured in FA-WIV and BPL-WIV samples were below the detection limit (n.d., not detectable). Results are presented mean±SEM (n = 20 eggs).
(TIF)

Figure S2 **Efficacy of CD8+ cell depletion in peripheral blood of mice after injection of YTS169 antibody.** On days 22, 23 and 24 after the start of experiment, mice were injected i.p. with a single dose of the depletion antibody. Subsequently, starting from day 1 post challenge, mice were injected with a single dose of the depletion antibody every 7 days. To monitor the efficacy of CD8+ cell depletion, blood samples were collected from mice on day 24 and then immediately prior to each subsequent antibody injection. As a control, blood was also sampled from mice that did not receive the depletion antibody. Isolated PBMCs were surface stained for CD8. To avoid multiple blood sampling from individual animals, samples were taken only from two mice per group at each time point. Representative flow cytometry plots are shown demonstrating the efficacy of CD8 cell depletion. Data is presented as the percentage of CD8+ cells within the total PBMC population.
(TIF)
