# Supplementary figures and images for: Induction of Heterosubtypic Cross-Protection against Influenza by a Whole Inactivated Virus Vaccine: The Role of Viral Membrane Fusion Activity
Source: PLoS One. 2012 Jan 27;7(1):e30898. doi: 10.1371/journal.pone.0030898 (PMC3267744; doi:10.1371/journal.pone.0030898)

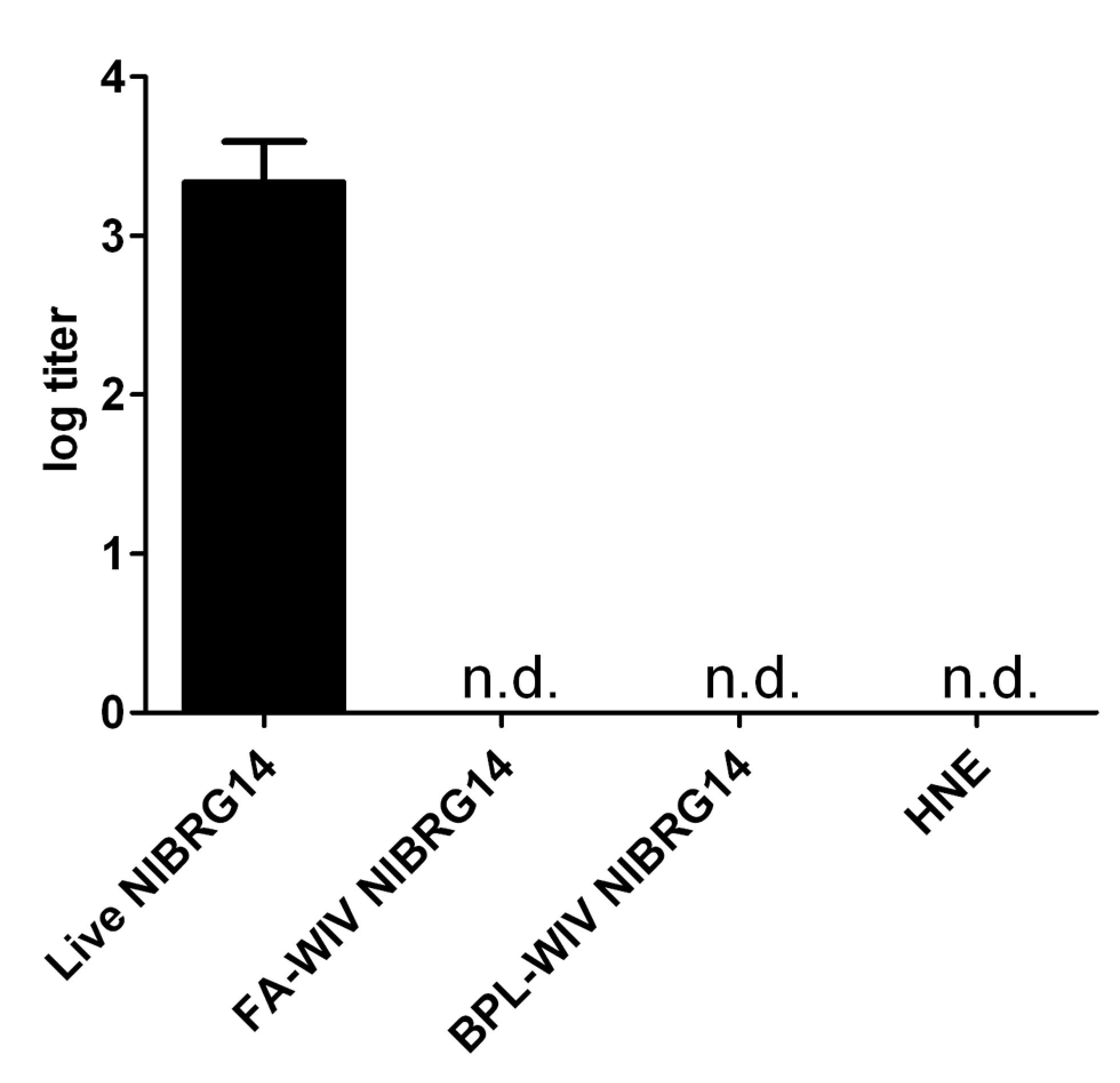

Supplement: Figure S1 — Assessment of viral inactivation status in the FA-WIV and BPL-WIV vaccines. Influenza virus inactivation was tested by performing serial passages on eggs. After the last passage, allantoic fluids were tested for the presence of replicative virus using the hemagglutination test. Virus titers measured in FA-WIV and BPL-WIV samples were below the detection limit (n.d., not detectable). Results are presented mean±SEM (n = 20 eggs). (TIF) [file pone.0030898.s001.tif]

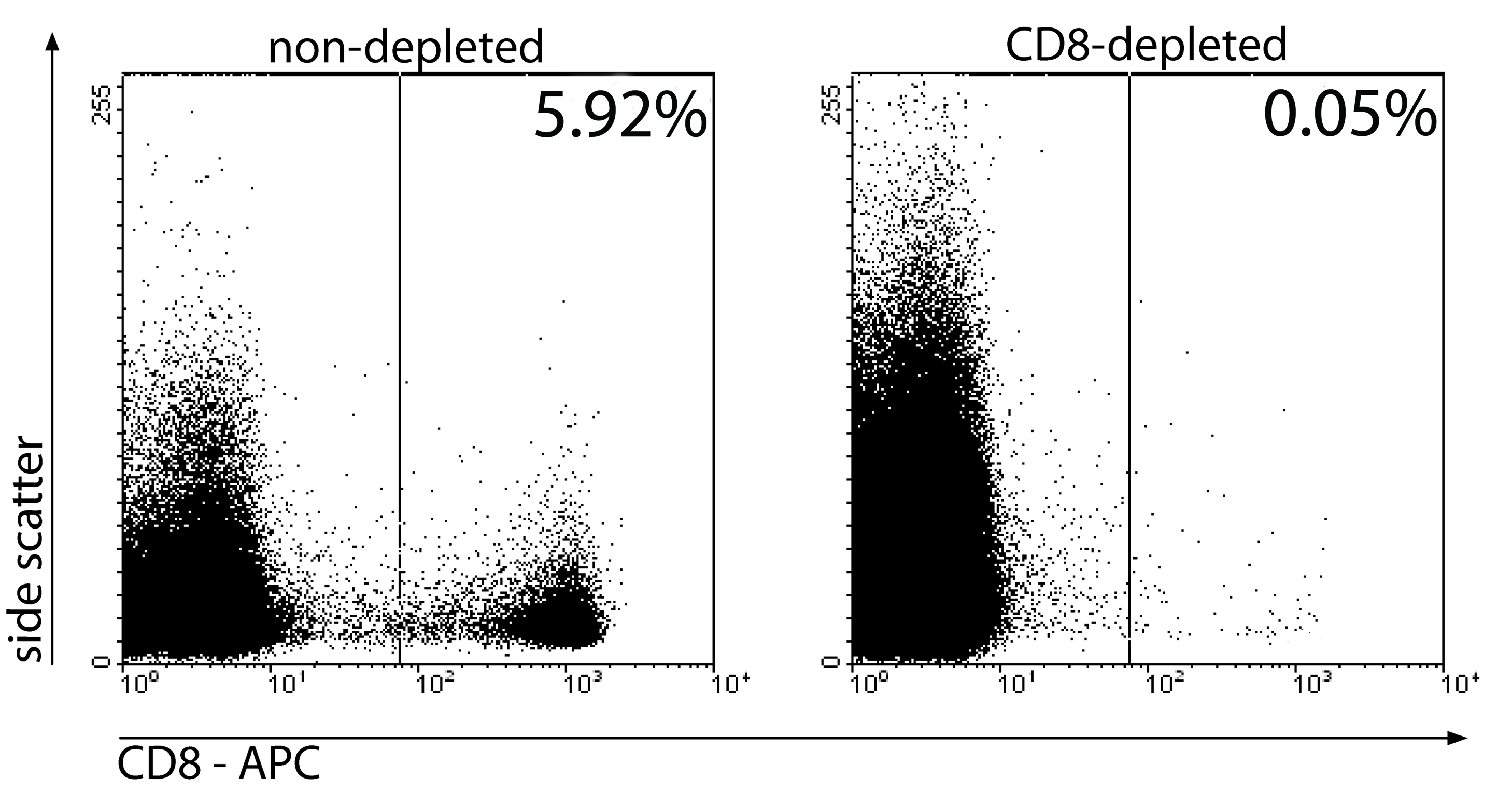

Supplement: Figure S2 — Efficacy of CD8+ cell depletion in peripheral blood of mice after injection of YTS169 antibody. On days 22, 23 and 24 after the start of experiment, mice were injected i.p. with a single dose of the depletion antibody. Subsequently, starting from day 1 post challenge, mice were injected with a single dose of the depletion antibody every 7 days. To monitor the efficacy of CD8+ cell depletion, blood samples were collected from mice on day 24 and then immediately prior to each subsequent antibody injection. As a control, blood was also sampled from mice that did not receive the depletion antibody. Isolated PBMCs were surface stained for CD8. To avoid multiple blood sampling from individual animals, samples were taken only from two mice per group at each time point. Representative flow cytometry plots are shown demonstrating the efficacy of CD8 cell depletion. Data is presented as the percentage of CD8+ cells within the total PBMC population. (TIF) [file pone.0030898.s002.tif]
